# Effects of Vitamin and Amino Acid-Enriched Hyaluronic Acid Gel on the Healing of Oral Mucosa: In Vivo and In Vitro Study

**DOI:** 10.3390/medicina57030285

**Published:** 2021-03-18

**Authors:** Elena Canciani, Riccardo Sirello, Gaia Pellegrini, Dolaji Henin, Mariachiara Perrotta, Marilisa Toma, Nataliya Khomchyna, Claudia Dellavia

**Affiliations:** Department of Biomedical, Surgical and Dental Sciences, University of Milan, Via Mangiagalli 31, 20133 Milan, Italy; riccardo.sirello@unimi.it (R.S.); gaia.pellegrini@unimi.it (G.P.); dolaji.henin@unimi.it (D.H.); mariachiara.perrotta1997@gmail.com (M.P.); marilisa.toma@unimi.it (M.T.); natalie.khom@gmail.com (N.K.); claudia.dellavia@unimi.it (C.D.)

**Keywords:** hyaluronic acid, vitamins, gel, impaired wound healing, oral mucosa, dental personalized medicine

## Abstract

*Background and Objectives*: Wound healing is a dynamic process that can be compromised in patients with chronic and metabolic conditions or unhealthy lifestyles. Numerous medical substances designed for topical use, charged with compounds that promote the healing process, have been developed to improve wound healing, especially in compromised subjects. The present study aimed to extend our understanding of the in vivo effects of a hyaluronic acid gel charged with amino acids (HAplus gel, Aminogam gel^®^ Errekappa Euroterapici spa, Milan, Italy) and study the in vitro effects of the same gel charged with additional substances in an attempt to optimize its formulation. *Materials and Methods:* In a randomized controlled split-mouth clinical and histological trial, HAplus gel was tested on the gingival tissue of the lower third molar post-extraction socket. The gingiva was collected at the time of extraction (T0) and ten days after the extraction (T1) to be histologically analyzed. During the second stage of the study, culture media with HAplus gel and vitamin C and E at different concentrations (TEST) were tested on human gingival fibroblasts and compared to the HAplus-enriched medium (HA-Control). *Results:* Histological and immunohistochemical analysis of collected gingiva showed higher microvascular density and collagen fibers organized in closely packed and well-oriented bundles in sites treated with HAplus gel. In the in vitro study, all TEST groups showed an increased viability from 24 h to 48 h. After 24 h, the viability percentage in all experimental groups was below 100% of the HA-Control, demonstrating a mild toxicity. After 48 h from seeding, the TEST groups’ viability grew significantly compared to HA-Control. *Conclusions:* These encouraging preliminary results suggest that the use of HAplus gel enriched with vitamins C and E may be beneficial in patients with conditions that impair soft tissue healing.

## 1. Introduction

Wound healing is a dynamic process that requires an efficient vascularization and new collagen matrix deposition. The growth of new vessels determines traction that induces remodeling of the extracellular matrix, potentiating neovessel responses. In clinical dental practice, rapid and integral healing of surgical wounds is fundamental to reducing the opportunity of infection and patient discomfort [1]. New medical substances for topical use, charged with compounds that support repairing mechanisms, have been developed to improve the healing process of damaged tissues [1,2].

Chronic and metabolic conditions such as diabetes and rheumatoid arthritis [3,4] or unhealthy lifestyles including smoking [5] play negative effects on angiogenesis and collagen fiber deposition/maturation, processes that are fundamental for tissue repair and regeneration [4,6,7,8,9]. Wound management of such patients remains an unresolved medical issue. Topical application of an agent that acts specifically on angiogenesis and collagen deposition could be beneficial.

Personalized medicine (PM) is an emergent medical model approach that makes use of novel developments in prevention, diagnosis, and treatment to offer the “right treatment for the right person at the right time” [10,11]. In the dental field, PM could find an important application in minimizing the onset and progression of oral chronic disease. It could also reduce the negative and detrimental effects that systemic conditions or chronic diseases have on oral health and wound healing. In these patients, wound healing can be promoted by the application of topical medication designed to stimulate neo-angiogenesis and collagen deposition.

Commercial preparation based on sodium hyaluronate (HA) with low molecular weight and a pool of synthetic collagen precursor amino acids (l-proline, l-leucine, l-lysine and glycine) (HAplus gel, Aminogam gel^®^ Errekappa Euroterapici spa) has been developed with a goal of improving connective tissue, and epithelium and mucosa healing [9,12,13]. HA is a largely used molecule because it creates a temporary structure for the deposition of extracellular matrix (ECM) proteins, triggers cell adhesion, proliferation and migration [13], and regulates vascular endothelial cell function [14,15,16]. Furthermore, it is involved in maintaining ECM resilience and tissue hydration. HAplus gel, tested in healthy patients, promoted the clinical healing of laser-induced wounds [17].

However, its effect on biological mechanisms, such as vascularization and collagen deposition in human healing mucosa, has not yet been investigated, reducing the possibility to improve the properties of the commercial preparations to personalize treatment in affected patients with conditions that impede wound healing.

A synergical mix of HAplus and antioxidants such as vitamin E (alpha-tocopherol) and vitamin C (ascorbic acid) could have a protective function against peroxidative damage caused by free radicals [18] and could stimulate cellular activity aiding the wound healing process [19].

The present study aimed to: (i) examine the biological effects of HAplus gel on wound healing in healthy patients (in vivo) in terms of neo-angiogenesis and collagen deposition; and (ii) optimize the gel formulation (in vitro) in order to personalize the treatment of patients with compromised tissue healing.

## 2. Materials and Methods

The study was divided in two phases, according to the in vivo and in vitro aims.

### 2.1. Phase I (In Vivo)

**Figure 1:**
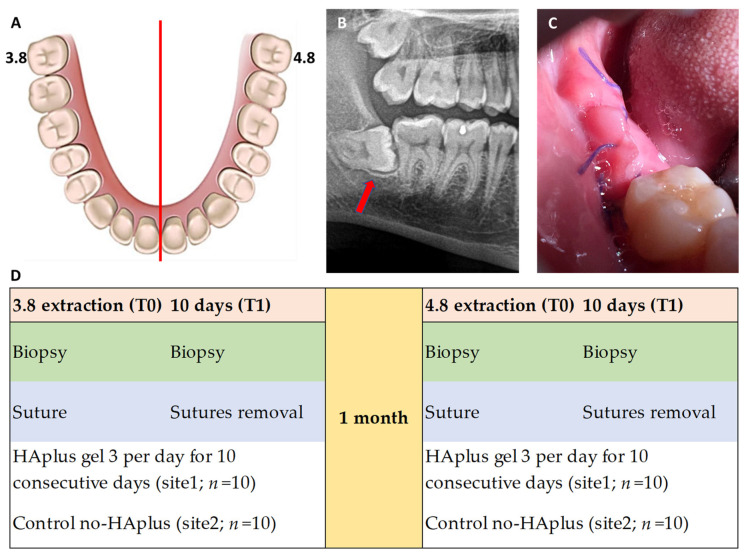
(**A**) Graphical representation of split-mouth design involving third molars 3.8 and 4.8; (**B**) red arrow indicates a mesioangular third molar at orthopantomography; (**C**) intraoral photograph shows post-extractive site at 10 days with flap closure via the Donati suture technique with consequent excesses of tissue to maintain tensile strength and create a stable gingival coverage for better healing of the alveolar socket. (**D**) Post-extraction treatment regimens tested: test sites were treated with HAplus (Aminogam gel^®^ Errekappa Euroterapici spa) gel, while control sites were not treated with any topical substances (no-HAplus).

**Study design and population.** This randomized controlled split-mouth clinical and histological trial included 10 adult patients who required the extraction of both inferior impacted third molars (3.8 and 4.8) (Figure 1A). Two post-extraction treatment regimens were tested: test sites were treated with HAplus gel, while control sites were not treated with any topical substances (no-HAplus).

*Inclusion criteria*

- Over 18 years old;
- Full-mouth plaque score (FMPS) and full-mouth bleeding score (FMBS) <15%.

*Exclusion Criteria*

- Systemic disease (tumors, diabetes, HIV, Hepatitis B-C);
- Smoking;
- History of keloids and diseases affecting collagen turnover;
- Pregnant or lactating women;
- History of bisphosphonate therapy;
- Periodontal and periapical disease of adjacent teeth.

**Population.** Sample size calculation was performed using α = 5% and the power of sample 95%. According to Canullo et al., 2020, the average microvascular density (MVD %) was from 8% to 12% with a standard deviation of 3%; therefore, the population of the study was calculated to be *n* > 7.35 [20].

Ten non-smoking subjects (7 women and 3 men) with a mean age of 34 years (from 25 to 41) were included in the study. A total of 40 samples were harvested and histologically analyzed in blind by two morphologists until the end of analysis: 20 samples were analyzed for the test group (10 HAplus-T0, 10 HAplus-T1), 20 samples for control group (10 no-HAplus-T0, 10 no-HAplus-T1).

**Treatment randomization.** The choice to treat 3.8 or 4.8 extraction sites with HAplus gel was decided by a coin toss.

**Informed Consent.** The study was conducted in accordance with the principles outlined in the Declaration of Helsinki and was approved by the Ethical Committee of the Università degli Studi di Milano, Milan (29/18 on 28 June 2018). All patients were informed about clinical procedures, post-extractive care, as well as the follow-up evaluations required for the study. All patients provided written informed consent for the collection of samples and their subsequent analysis. All clinical procedures and measurements were carried out by a private practitioner (R.S.).

**Surgical procedure.** In each patient, both teeth were extracted following the same surgical procedure; 3.8 was extracted first, and 4.8 was extracted 30 days later to ensure the patient’s recovery. All patients had impacted third molars with mesial inclination of the crown (Winter classification 1 and 2) (Figure 1B).

Following local anesthesia, using articaine with epinephrine 1:100,000 (Pierrel Pharma S.P.A., Cantù, Italy), a surgical incision was performed to expose the impacted tooth. A soft tissue biopsy was taken and treated for laboratory analysis (T0). After the extraction, the flaps were sutured with Vycril 3/0 (Aragò Violeta, Barcelona, Spain) following the Donati technique, which enables maintaining of the tensile strength and creates a stable gingival coverage for better alveolar healing [21]. This suturing technique also causes an excess of soft tissue (Figure 1C).

**Post-surgical treatment.** Following the extraction, patients were prescribed HAplus gel for the test sites, to be used 3 times per day for 10 consecutive days. No gel was prescribed for the control sites (no-HAplus) (Figure 1D).

An antibiotic therapy (Amoxicillin 1 gr/3 times a day for 7 days) was also prescribed to each patient for 7 days after each extraction, while an analgesic (ibuprofen 600 mg) therapy was given only if needed (Table S1). Mouthwashes or other oral topical substances were suspended.

In both groups, sutures were removed 10 days after the extraction, and gingivoplasty to remove excess soft tissue was performed. Collected samples of the mucosa were treated for laboratory analysis (T1) (Figure 1D).

**Histological Processing.** Immediately after harvesting, the gingival specimens were immersion-fixed in 10% formalin/0.1 M phosphate buffer saline (PBS, pH 7.4) for 24 h at room temperature, then dehydrated in increasing concentrations of ethanol (70 to 100%), clarified in xylol and finally embedded in paraffin. Then, 5 µm serial sections of paraffin-embedded tissue blocks were obtained, mounted on 3-amino-propyl-trietoxi-xilane coated slides, hydrated in decreasing concentrations of xylol and ethanol (100 to 70%) and immersed in distilled water. The representative sections were stained with Carazzi’s hematoxylin and eosin (H&E) to analyze the morphology of the epithelium, overall connective tissue structure, and inflammatory infiltrate, and with Sirius Red/Picric Acid 0.1% (Sigma Aldrich, Milan, Italy) to detect collagenous proteins in the connective portion.

The microvessel content was assessed by means of immunostaining performed on 4 slides (2 marked and 2 controls per site) with mouse monoclonal CD31 antibody (Leica Biosystem, Milan, Italy) against the endothelium of blood vessels in accordance with the standard protocol of UltraVision^TM^ Quanto Detection System HRP DAB kit (ThermoFischer Scientific, Milan, Italy) [20].

**Histomorphometry.** All the histological slides were scanned by a high-resolution digital scanner (Aperio Scan Scope System CS2, Leica Biosystem, Milan, Italy) at 40× resolution. Digital slides were navigated from 5× to 400× magnification.

A histomorphometric analysis of the H&E slides was performed at a total magnification of 400×, following the indications of ISO-10993-6:2007 annex E:

— “cell type/response”—evaluation of necrosis and inflammatory infiltrate.
— “tissue response”—analysis of microvascular density (MVD), fibrosis and fatty infiltrate in the healing area.

The MVD was qualitatively evaluated on the H&E slides and quantitatively on the slides with CD31 antibody (JC/70A, ab9498, abcam, Cambridge, UK) marked vessels (negative control: no primary antibody; positive control: tonsil). The immunohistochemistry of CD31 antibody was used to calculate the percentage of microvessels in the connective tissue by stereology-based method on the slides scanned with an Aperio Scan Scope System CS2 (Leica Biosystem, Milan, Italy). A customized digital counting grid was employed to evaluate the MVD of each tissue slide by means of histomorphometric analysis. More specifically, the intersection points that fell on the vessels were manually counted, and the ratio between test points and total points of the grid that fell on the overall connective tissue was calculated and expressed as a percentage value [22].

The Sirius Red-stained sections were analyzed by a polarized light microscope (Eclipse E600) equipped with a calibrated digital camera (DXM1200, Nikon, Tokyo, Japan). Newly deposited collagen was observed under polarized light to estimate collagen quantity and evaluate fibers orientation using a dedicated navigation software (Photoshop CS3, Inc., New York, NY, USA). The connective tissue was digitally selected out of the whole gingival section and the ratio of Sirius Red-stained surface indicating the collagen area (red pixel) to the total connective area (total pixel of the connective tissue) was calculated and expressed as a percentage [22].

**Statistical Analysis.** Descriptive and inferential statistics were carried out on the data obtained from CD31 immunostaining and Sirius Red analyses by means of the Ky Plot 5.0 software, Informer Technologies, Inc., New York, NY, USA.

The data obtained at T1, both HAplus and no-HAplus, were normalized on relative T0 data for both Sirius Red and CD31 immunostaining analyses. An inferential statistical analysis was developed on normalized data between HAplus and no-HAplus sites for each parameter using the Wilcoxon signed-rank test for paired data without continuity correction and with a level of significance of 5% (*p* < 0.05). Lastly, mean values and standard deviations were performed on normalized data of each parameter.

### 2.2. Phase II (In Vitro)

The second phase aimed to assess the formulation of a new gel HA-plus base, enriched with vitamin C and vitamin E. Optimal concentration of vitamins was evaluated in vitro according to ISO standards. The most favorable concentration was determined choosing a lower cellular toxicity and higher proliferation between timepoints.

**Cell culture and design of the study.** The experiments were conducted when the human gingival fibroblast culture (HGF) (ATCC^®^ PCS-201-018™) passage grew to 80% confluence between fourth and fifth.

All experiments were performed at 37 °C in a 5% CO_2_ humidified incubator using, for each ml of culture medium (DMEM, high Glucose, Euroclone, Milan, Italy), 10% heat-inactivated fetal bovine serum (FBS) (Euroclone, Milan, Italy), supplemented by 100 U penicillin (Invitrogen), 100 μg streptomycin (GIBCO), 2.5 μg amphoterycin B (Sigma Aldrich, Milan, Italy) [23]. To obtain the HA-medium, 1% of HA-plus was added (HA-medium) to the culture medium, according to Colella et al.’s 2012 study [24].

**Vitamin preparation.** The percentages of vitamins were estimated following the Food and Drug Administration’s recommended intake for a 70 kg man. The vitamin solution was obtained dissolving 1.4 μg/mL of hydro-soluble vitamin C (L-ascorbic acid; Sigma, St. Louis, MO, USA) and 0.14 μg/mL of liposoluble vitamin E (α-Tocopherol; Sigma, St. Louis, MO, USA) using a solubilizer (Kolliphor RH 40, Sigma Aldrich, Milan, Italy, at 8% of the solution) in DMEM. The solution was prepared, mixed by vortex, and kept away from light sources in a dark bottle, due to the photosensitivity of the vitamins. The solution’s pH was maintained close to 7.4, stocked at 4 °C.

**Viability assay.** Different concentrations of the vitamin solution were added to HA-medium, as reported below:

— HA-VIT-1: HGF in HA medium with vitamin solution at 1%;
— HA-VIT-2.5: HGF in HA medium with vitamin solution at 2.5%;
— HA-VIT-5: HGF in HA medium with vitamin solution at 5%;
— HA-Control: HGF in HA medium.

To test cell viability, 7 × 10^3^ cells were seeded in 24-well multi-well plates. Cell viability was tested at 2 different time points (24 and 48 from seeding) using Alamar Blue^®^ (Thermo Fisher Scientific, Milan, Italy), a test that allows the determination of a proliferation curve related to the metabolic cellular ability. The color changing of the culture medium revealed an active cellular metabolism, which was monitored and read by a spectrophotometer (Glo Max Discover, Promega Corporation, Madison, WI, USA). For each group, a proliferation curve was created, and the percentage of cell proliferation was calculated in relation to untreated sample proliferation, imputing the values in an algorithm, as described by the manufacturer’s protocol.

**Data Analysis.** The experimental groups were set and tested in triplicates, in the dark, at 24 h and 48 h. For each TEST group (HA-VIT-1, HA-VIT-2.5, HA-VIT-5), a proliferation curve was drawn and the percentage of cell proliferation at 24 h and 48 h was calculated, comparing it to HA-Control using AlamarBlue^®^ assay (Thermo Fisher Scientific Inc, Milan, Italy) [25]. The data were expressed as the mean and standard deviation of cells viability.

## 3. Results

### 3.1. Phase I (In Vivo)

**Figure 2:**
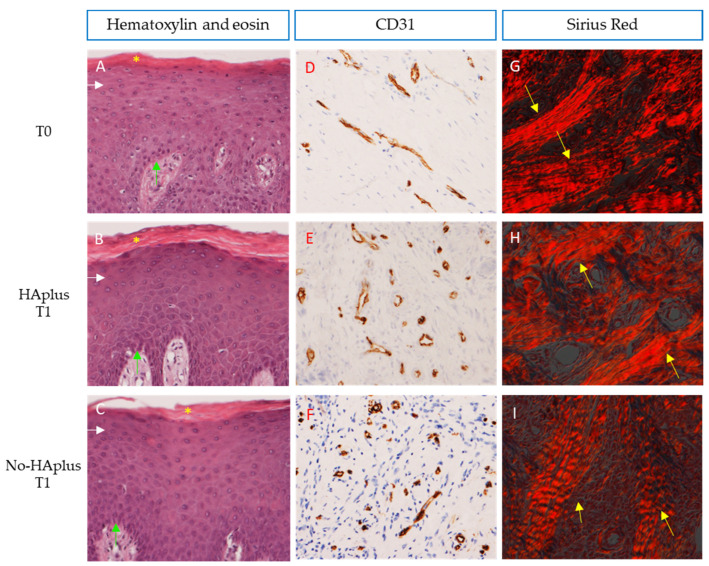
**Gingiva:** (**A**–**C**) Sections demonstrate the integrity and normal stratification of the epithelium at the healing site. Pluri-stratified squamous epithelium with ortho- and para-keratinization is depicted in pink and indicated with yellow asterisks, while basal (light blue arrows) to granular layers (white arrows) of epithelium are shown in purple. Carazzi’s H&E, total magnification 200×. (**D**–**F**) Sections stained with CD31 in brown demonstrate differences in microvascular distribution for each group. Immunostaining anti-CD31, total magnification 200×. (**G**–**I**) Sections highlighted under polarized light showing the orientation and distribution of collagen fibers in red. More evident collagen bundles are indicated with yellow arrows. Sirius red/Picric Acid, total magnification 200×.

**Morphological assessment.** Histological observations of H&E sections following the indications of ISO-10993-6:2007 annex E showed no necrotic areas, no fatty-cell infiltration, and no fibrosis in both groups and timepoints. Sparse inflammatory infiltrate was observed in the connective tissue of two patients at T0. In all samples, the epithelium presented a normal stratified structure and keratinization status (Figure 2A–C).

**Figure 3:**
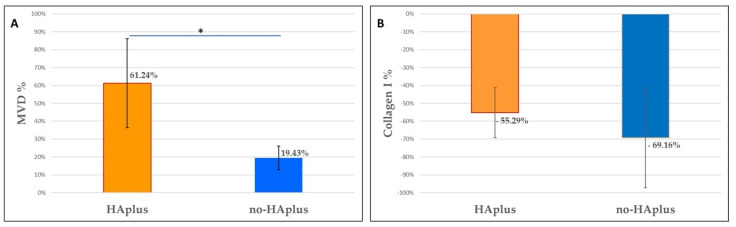
**Histomorphometrical analysis.** (**A**) Graphics show the increase in microvascular density (MVD) and (**B**) decrease in collagen content (Collagen I %) that occurred in both groups (HAplus, no-HAplus) after 10 days of healing. The MVD increases in HAplus sites resulted significantly higher than in no-HAplus sites (Wilcoxon signed-rank test) (*p* < 0.001). Data were normalized on relative T0 values for both MVD and % Collagen.

**Microvascular density.** Sections stained with CD31 showed differences in microvascular distribution, between groups and timepoints (Figure 2D–F). At T0, MVD was similar in both groups. At T1, the MVD had increased in both groups and was significantly higher than at T0 in both groups (Wilcoxon signed-rank test, *p* < 0.001). However, this increase was significantly higher in HAplus than in the no-HAplus group (Wilcoxon signed-rank test on normalized data between groups, *p* = 0.013) (Table 1, Figure 3A).

**Collagen content.** At the observation under polarized light, both groups showed a lower fiber organization at T1 compared to T0 (Figure 2G–I). At T0, collagen fibers were organized in closely packed and well-oriented bundles. At T1, connective tissue of the no-HAplus group was characterized by a disorganized grid of thin collagen fibers, while in the HAplus group, fibers appeared oriented in a more parallel manner that in the no-HAplus samples and were alternated with areas of thin crossed collagen fibers. No difference was found in collagen content between groups at both timepoints. Values at T1 resulted significantly lower than at T0 in both HAplus and no-HAplus sites (Wilcoxon signed-rank test, *p* < 0.001), and no difference in the decrease rate was found between post-op treatments (Table 2, Figure 3B).

### 3.2. Phase II (In Vitro)

**Figure 4:**
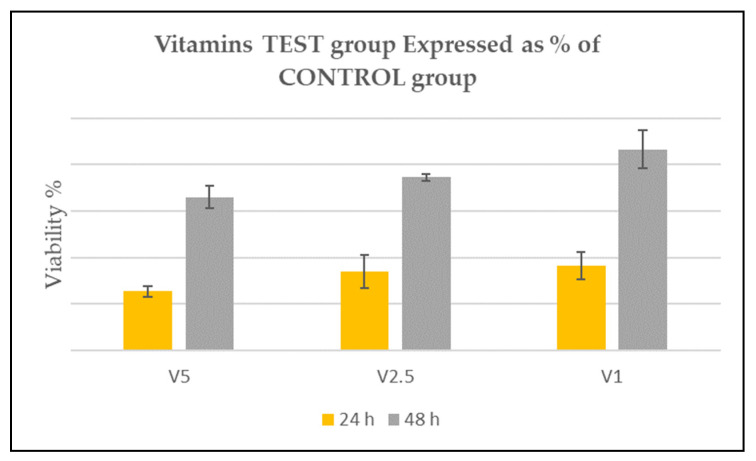
**Vitamin test groups.** Graphic shows the viability of the TEST groups. According to ISO 10993-5, after 24 h, a reduction up to 50% was considered slightly toxic as evident in HA-VIT-5; up to 20% of the viability was considered mildly toxic as in HA-VIT-2.5 and HA-VIT-1. After 48 h, viability increased, overcoming the HA-Control value (100%) in all experimental groups.

**Vitamin concentration in HA culture**. In all TEST groups, viability increased from 24 h to 48 h. All samples presented the same trend. After 24 h, the viability percentage resulted in less than 100% of the HA-Control, resulting to be mildly toxic in all experimental groups. After 48 h from seeding, the TEST groups’ viability grew significantly compared to HA-Control, suggesting an advantageous effect of the vitamins on cell viability. In all cases, the increment result was statistically significant, especially in HA-VIT-1 group (Figure 4).

## 4. Discussion

In the present study, the in vivo part was designed to deepen our understanding of the biological effects of HAplus gel on wound healing in healthy patients (in vivo) in terms of neo-angiogenesis and collagen deposition. Based upon the data obtained, the in vitro part was conducted to optimize the gel formulation (in vitro) for the treatment for patients with compromised tissue healing. The main purpose of the present study was to formulate a gel for topical use to be applied in patients with impaired oral wound healing.

Firstly, to conceptualize the new medical device, a clinical study was set in healthy patients to assess the effects of a commercial HA/amino acid gel (HAplus) on neovascularization and collagen fiber formation (phase I) after oral surgery. These two physiological activities were selected for the analysis due to their central role in mucosa healing. In fact, non-healing wounds are often characterized by poor vascular network [2] and altered collagen production. Furthermore, the formation and orientation of collagen fibers influences the growth rate and direction of neo-vessel migration through the healing matrix [26]. From histological data of the study’s phase I, the healed oral mucosa in both groups was characterized by a higher vascularization and a lower collagen content than pristine samples, indicating that an incomplete stage of healing is reached by the connective tissue 10 days post-surgery. In sites treated with HAplus gel, the MVD was significantly higher and collagen fibers appeared more organized than in untreated sites, indicating a more favorable healing activity in treated experimental sites [27]. From these data, it can be assumed that in healthy patients the topical application of HA and amino acids on wounds may exert a beneficial effect on stromal vascularization, unlike the collagen deposition. The obtained results on vascularization seem to confirm the crucial role of HA in the regulation of the angiogenic process, by acting on endothelial cell function [28], migration [29], and sprout formation [30]. Furthermore, in a 2020 clinical study, Çankaya et al. observed that topical HA application on free gingival graft donor and recipient sites during the early phases of wound healing allows the formation of a well-vascularized layer, which acts as a barrier against tissue tensions by functioning as a scaffold between the recipient bed and gingival graft [31]. Numerous in vitro and animal model studies have reported the positive effects of amino acid mixtures on wound healing [32,33]. For example, proline and its precursors were reported to increase collagen synthesis in human fibroblast cells [32], and leucine supplementation was reported to have an anabolic effect on protein metabolism in skin wounds in rabbits [34]. Furthermore, synthesis of collagen II seemed to be stimulated by a wide range of different concentrations of glycine, proline, and lysine in a culture of bovine chondrocytes [35].

In the present study, the data from phase I showed no differences regarding the amount of collagen fibers between treated and untreated sites, which may indicate a similar collagen protein synthesis rates by cells. Fibroblasts play a crucial role in wound healing closure [36]; therefore, a topical gel with stimulating properties for patients with impaired wound healing should contain biomolecules that improve the viability and activity of these cells.

The adding of antioxidants to HAplus gel aimed at reducing the oxidative stress associated with various disease conditions [37] and to stimulate fibroblast cells to depose more collagen fibers [23]. Barbosa et al. observed that the supplementation of antioxidants, such as vitamin E and C and the mineral zinc, seemed to enhance protection against oxidative stress, reducing wound healing time in children [37]. Scholl et al. (2001) reported that a mix of vitamins E and C and zinc performed a key role in collagen synthesis, growth, and cell replication, and also in immune system function during the healing process [38].

Therefore, the second part of the present study aimed at evaluating the viability of HGF in the presence of HAplus supplemented with vitamins C and E. We chose vitamin C and vitamin E as active agents due to their antioxidant [37,38] and collagen-stimulating properties [39]. Vitamin C is a strong water-soluble antioxidant and a powerful reducing agent, able to regulate the expression of the procollagen gene, stimulating collagen synthesis, and fibroblast and osteoblast differentiation and proliferation [39,40,41]. This vitamin is often used to supplement fibroblast culture medium to maintain the functionality and the differentiation of the cells [24]. In clinical practice, vitamin C-based gel applications are indicated and recommended for people with collagen turnover deficit [39,40]. Vitamin E is a lipid-soluble antioxidant, critical for cell membrane maintenance, and is important for the improvement of hard [42] and soft tissue [39] healing. In phase II, the HAplus plus vitamins formulation was set, following ISO-10993 standard, in order to achieve optimal and non-toxic concentrations of vitamins for fibroblast viability. The experiments demonstrated that all vitamin concentrations allowed an increased viability between timepoints. The supplementation with 2.5% and 1% vitamins resulted to be less cytotoxic for HGF, suggesting that the initial viability reduction in the experimental groups could be attributed to cell adaptation in the vitamin medium. At 48 h, in fact, in all experimental cases, the cells started to metabolize the substances and to restore the viability, overcoming the HA-Control, suggesting a potential beneficial role for tissues in the active phase of healing, especially in subjects with impaired collagen deposition and wound healing.

## 5. Conclusions

In conclusion, this clinical study demonstrated that the application of HAplus gel in healthy patients provides beneficial effects on oral wound healing in terms of increased vascularization and improved collagen fiber organization. Additionally, HAplus gel supplemented with low concentration of vitamins showed positive effects on gingival fibroblasts in culture. These encouraging preliminary results may suggest the use of HAplus gel enriched with vitamins in patients with diseases or conditions that impair soft tissue healing.

This study format suggests a model for treatment personalization, which could be optimized by the use of the most recent technologies, such as nanotechnologies, able to specifically act on impaired biological functions through drug delivery and mechanical support.

## Figures and Tables

**Table 1:** This table reports the mean values and standard deviations of microvascular density (MVD) in both groups (HAplus: test group, no-HAplus: control group) at T0 (baseline) and T1 (10 days after tooth extraction), obtained using histomorphometrical evaluation. The microvascular density increased from T0 to T1 by about 5% in the HAplus group, and 1.5% in the no-HAplus group.

MVD	T0	T1	
HAplus	7.00% *±* 1%	11.91% *±* 3%	*p* < 0.001
no-HAplus	7.18% *±* 1%	8.62% *±* 2%	*p* < 0.001
	ns	*p* = 0.013	

**Table 2:** This table reports the mean values and standard deviations of the collagen content (% Collagen) in both groups (HAplus: test group, no-HAplus: control group) at T0 (baseline) and T1 (10 days after tooth extraction), obtained using histomorphometrical evaluation. The presence of collagen fiber content decreased from T0 to T1 by about 50% in both groups.

Collagen Content	T0	T1	
HAplus	38.96% *±* 5%	18.92% *±* 7%	*p* < 0.001
no-HAplus	40.98% *±* 8%	18.45% *±* 5%	*p* < 0.001
	ns	ns	

## Data Availability

The data that support the findings of this study are available from the corresponding author upon reasonable request.

## Supplementary Materials

The following are available online at https://www.mdpi.com/1648-9144/57/3/285/s1, Table S1: The table shows gender, age, impacted tooth class following Winter classification and drug administration. Amx = Amoxicillin; NSAIDs = Ibuprofen 600 mg administered in both study groups.
