# Supplementary material for: Effects of Vitamin and Amino Acid-Enriched Hyaluronic Acid Gel on the Healing of Oral Mucosa: In Vivo and In Vitro Study
Source: Medicina (Kaunas). 2021 Mar 18;57(3):285. doi: 10.3390/medicina57030285 (PMC8003116; doi:10.3390/medicina57030285)
Supplement: Supplementary file 1 [file medicina-57-00285-s001.pdf]

Table S1. The table shows gender, age, impacted tooth class following Winter classification and drug administration. Amx = Amoxicillin; NSAIDs = Ibuprofen 600mg administered in both study groups.

| | | | HAplus | | No-HAplus | |
|---|---|---|---|---|---|---|
| **PATIENT** | **GENDER** | **AGE** | **IMPACTED TOOTH (Winter Classification)** | **DRUG ADMINISTRATED** | **IMPACTED TOOTH (Winter Classification)** | **DRUG ADMINISTRATED** |
| 1 | F | 26 | Class 1 | Amx; | Class 1 | Amx |
| 2 | F | 35 | Class 1 | Amx; NSAIDs | Class 1 | Amx; NSAIDs |
| 3 | F | 40 | Class 2 | Amx; NSAIDs | Class 1 | Amx; NSAIDs |
| 4 | F | 28 | Class 1 | Amx | Class 1 | Amx; NSAIDs |
| 5 | F | 36 | Class 2 | Amx; NSAIDs | Class 2 | Amx; NSAIDs |
| 6 | F | 25 | Class 1 | Amx; NSAIDs | Class 1 | Amx; NSAIDs |
| 7 | F | 41 | Class 1 | Amx | Class 1 | Amx |
| 8 | M | 39 | Class 1 | Amx | Class 1 | Amx |
| 9 | M | 33 | Class 2 | Amx; NSAIDs | Class 2 | Amx; NSAIDs |
| 10 | M | 37 | Class 1 | Amx; NSAIDs | Class 1 | Amx; NSAIDs |
